# Gearing up to improve exclusive breastfeeding practices in South Africa

**DOI:** 10.1371/journal.pone.0265012

**Published:** 2022-03-10

**Authors:** Debbie Vitalis, Chantell Witten, Rafael Pérez-Escamilla

**Affiliations:** 1 School of Public Health, Yale University, New Haven, Connecticut, United States of America; 2 Faculty of Health Sciences, University of the Free State, Bloemfontein, South Africa; Christiana Care/University of Delaware, UNITED STATES

## Abstract

South Africa has one of the lowest breastfeeding rates on the African continent. Globally, just 44% of infants are breastfed soon after birth, and 40% of those less than six months old are exclusively breastfed. To improve infant nutrition by 2025, the United Nations established targets to eliminate malnutrition and increase exclusive breastfeeding (EBF) rates to at least 50%. Despite the WHO Code regulations endorsed by the World Health Assembly since 1981, breaches continue to be prevalent due to a combination of weak implementation, monitoring and enforcement in low-to-middle income countries. Over the years, infant formula sales in LMICs (including South Africa) have skyrocketed contributing to excess infant morbidity and mortality. To that end, the specific aims of this study was to gain an understanding of priority actions and strategies necessary to improve breastfeeding outcomes in South Africa in the context of the HIV pandemic. The team used a qualitative study design based on a semi-structured interview guide. The guide consisted of eight open-ended questions addressing the WHO HIV-related infant feeding guidelines, the WHO International Code of Marketing of Breastmilk Substitutes, political will, and advocacy. Of the 24 individuals contacted, 19 responded and 15 agreed to participate. The Breastfeeding Gear Model guided the thematic analysis. The three main themes identified were 1) WHO guidelines on HIV and infant feeding, 2) Improving exclusive breastfeeding, and 3) Advocacy. Key informants identified issues that need to be addressed to improve breastfeeding outcomes in South Africa. Strong political will is a key ingredient to harness the resources (human, financial) needed to implement, monitor, and act against Code violators. South Africa and other countries with similar challenges should consider using the WHOs Network for Global Monitoring and Support for Implementation of the International Code of Marketing of Breast-milk Substitutes and Subsequent relevant World Health Assembly Resolutions (NetCode) methodology.

## Introduction

South Africa has one of the lowest breastfeeding rates on the African continent. Globally, just 44% of infants are breastfed soon after birth, and 40% of those less than six months old are exclusively breastfed [1, 2]. To improve infant nutrition by 2025, the United Nations established targets to eliminate malnutrition and increase exclusive breastfeeding (EBF) rates to at least 50% [3].

The process of breastfeeding and the nutrients, immunological components and other bioactive substances in breastmilk are essential for an infant’s growth, health, and development [1, 4, 5]. Breastfeeding reduces the risk of infectious diseases and obesity including promoting cognitive development in children [4, 6–8]. Moreover, women who breastfeed have a reduced risk of developing chronic diseases such as breast and ovarian cancer, hypertension, and type 2 diabetes [4, 9, 10]. The World Health Organization (WHO) and the United Nations Children’s Fund (UNICEF) recommend exclusive breastfeeding of infants from birth to six months, followed by introduction of complementary foods with continued breastfeeding for at least two years [1].

There have been several iterations of WHOs guidelines on infant feeding in the context of HIV as the science has evolved. In 2001, women were advised to abstain from breastfeeding in higher income countries because of the possibility of transmission of the virus through breastmilk in the context of relatively low infant mortality rates [11]. In 2006, the guidelines stated that early cessation of breastfeeding, i.e. prior to 6 months was not necessary [12]. A subsequent change in 2010 recommended that women on antiretroviral medication (ART) could exclusively breastfeed for the first six months with continued breastfeeding for a year [4, 13]. The most recent iteration in 2016 extended breastfeeding to at least 24 months as long as women are receiving ART [1].

South Africa embraced WHOs guidelines as they were issued and adapted their local policies accordingly. In 2011, EBF was recommended for all women regardless of their HIV status [14, 15]. Consistent with the Code, the declaration also discouraged distribution of formula at public health facilities except for medical reasons [2, 14].

Despite the WHO Code regulations endorsed by the World Health Assembly since 1981 [16], breaches continue to be prevalent due to a combination of weak implementation, monitoring and enforcement in low-to-middle income countries [17–19]. Over the years, infant formula sales in LMICs (including South Africa) have skyrocketed contributing to excess infant morbidity and mortality [17, 18, 20, 21].

A recent systematic scoping review by the authors on breastfeeding inequities in South Africa [22] identified free distribution of infant formula and aggressive marketing tactics by the formula industry as negatively impacting EBF practices among both HIV-negative and positive women in South Africa. The review also highlighted that enforcement of the WHO Code is necessary but not sufficient to improve breastfeeding outcomes as other factors such as social support and quality maternity care services are needed by women antenatally and post-delivery. We concluded that mixed-methods research, particularly in-depth interviews with key informants from major sectors (government, academia, civil society) were needed to identify actions that need to be prioritized and strategies to empower women to breastfeed more successfully in South Africa. To that end, the specific aims of this study was to gain an understanding of priority actions and strategies necessary to improve breastfeeding outcomes in South Africa in the context of the HIV pandemic.

## Methods

### Study design

The team used a qualitative study design based on a semi-structured interview guide. The guide consisted of eight open-ended questions (with probes) which included topics on the WHO HIV-related infant feeding guidelines, International Code on Marketing of Breastmilk Substitutes, political will, and advocacy (Table 1). It was developed and used in English with all participants. Topics for the guide were developed by DV based on the authors’ systematic scoping review [22], and reviewed by RPE taking into account his experience in the field. The study was approved by Yale University’s Institutional Review Board.

**Table 1:** Interview guide: Domains and questions.

Domains	Questions
**Opening/Background**	Can you please tell me about your background and current/previous role as it pertains to working with the WHO Code and HIV-related infant feeding guidelines in South Africa?
**Infant feeding guidelines**	How do you think the WHO HIV-related infant feeding guidelines over the years affected EBF/breastfeeding practices in South Africa?
**Legislation/Policies**	South Africa has made important strides in legislating the Code, so why has that not improved EBF/breastfeeding behavior?
**Political will/Government policies**	From your perspective, has the Government committed to enforcement of the Code? Please explain.
**Provider training/Support**	What role should health providers in-service or pre-service training play with reference to the a) The Code and b) HIV-related infant feeding guidelines?
**Advocacy**	In what ways can EBF/breastfeeding advocates advance the a) implementation and b) enforcement of the Code?
**Social/Other Support**	How can women be supported to improve EBF practices?
**Barriers**	What barriers at the a) individual, b) health facility and c) community levels need to be addressed to improve breastfeeding outcomes?

### Participants and data collection

Using snowball sampling, an initial pool of potential participants was purposively identified by one author (RPE) to ensure that they were subject-matter experts on the WHO Code and/or the Infant feeding guidelines as they pertain to South Africa. Subject matter experts were persons with extensive knowledge of or at least 5 years of experience implementing infant feeding guidelines and the World Health Organization’s Code of Marketing of Breastmilk Substitutes in South Africa. These initial invitees identified others who were eligible for the study. An email invitation was sent out to potential participants, followed by two additional emails for non-responders. Potential participants indicated their preferred date and time for the interview. All participants provided verbal consent to be interviewed which was audio recorded on Zoom. Data collection occurred between April 2021 and May 2021.

### Transcription and information saturation

Interviews were audio recorded with interviewees’ permission and transcribed by a HIPAA compliant transcription company (GMR Transcription, www.gmrtranscription.com). Transcripts were de-identified to ensure anonymity of participants. Interviews continued until we achieved saturation (when no new ideas or information was introduced).

### Data analysis

Thematic analysis was used to analyze the data. The team developed a codebook using a priori and emergent codes. Transcripts were managed and analyzed in Microsoft Word and NVivo. The Breastfeeding Gear Model (BFGM) was used as the framework for the analysis [23]. The BFGM framework describes the mechanism for successful breastfeeding. Several key elements or “gears” need to be in-sync to achieve breastfeeding success on a large scale. Advocacy is required to drive political will needed to ratify legislation and policies for health facilities and within communities. These policies are expected to churn the necessary resources to support staff and program development. Finally, research and evaluation and strong evidence-informed coordination are required to inform goal setting and provide feedback [23].

Our thematic analysis included initial coding by both authors (RPE, DV) using Microsoft Word. We independently coded three transcripts to arrive at consensus on the initial codes. The final codebook was the product of an iterative process and included identifying themes and subthemes. We achieved thematic saturation as the final interview yielded no new themes [24, 25]. Once the codebook was refined through an iterative process, all interviews were re-coded on NVivo v.12 qualitative software by one author using a consistent coding format and the final codebook.

## Results

### Descriptive findings

Of the 24 experts invited to participate, 19 responded and 15 participated. Reasons for declining to participate included lack of expertise on the Code, left South Africa years ago and not focusing on nutrition issues and being too busy. There was broad representation from academia, civil society, United Nations’ organizations, and the Government of South Africa, as persons were either currently or formerly associated with these institutions. Years of experience on the topics ranged from 5 years to 35 years.

### Themes

The three main themes identified were 1) WHO guidelines on HIV and infant feeding, 2) Improving exclusive breastfeeding, and 3) Advocacy (Table 2).

**Table 2:** Themes and quotations.

Theme	Sub-theme	Quote
**Theme #1:** WHO guidelines on HIV and infant feeding	Sub-theme 1a –Poor Code enforcement	At that time, WHO guidelines were that countries could choose to provide free formula or to support exclusive breastfeeding–Interviewee #5
	Sub-theme 1b –Political will to enforce the Code	The ongoing promotion and extensive and increasing promotion of the breastmilk substitutes, also, had a very strong negative effect on breastfeeding.–Interviewee #15
		It created this impression that formula milk is endorsed by healthcare professionals. It definitely had a spillover effect where people who were HIV positive or negative and may have decided to breastfeed or not, they were influenced by this seeing of infant formula and seeing or thinking that healthcare workers are saying it’s superior to breastfeeding and then that spillover effect of people who would have breastfed then decided to opt for formula feeding–Interviewee #12
		There are pockets of government that are very committed and there are pockets of government that are not.–Interviewee #14
		The regulations are relations is from birth to three years. So, they would now develop products for children above three years and then they would advertise it on Facebook, on Twitter.–Interviewee #1
**Theme #2:** Improving EBF/breastfeeding	Sub-theme 2a –Maternity protection and support	We know that going back to work early means that breastfeeding is likely to stop. So, to me maternity protection, paid breastfeeding, paid maternity leave and breastfeeding support in the workplace is really important.–Interviewee #7
	Sub-theme 2b –Behavior-change communication (BCC) campaigns	I think the community-based support is critical, and that’s very much lacking at the moment. I would say that would be the most important first step is ensuring that because we have such early discharge from hospital that there is some kind of link between the formal facility and the home setting.–Interviewee #8
	Sub-theme 2c –Training and Research	I think it also comes back to getting those influencers on board in the community. If you just target the mother, the woman’s immediate family, which could be a spouse of some sort or a partner of some sort, grandma, or mother-in-law, those are two key players who live in the community, and if you get their support, then you’re already affecting change in community.–Interviewee #14
**Theme #3:** Advocacy	Sub-theme 3a –Actions by civil society and other stakeholders	We had one incident, where a company had a promotion at a bus stop or a taxi. And the civil society was so involved with this and the company removed it with immediate effect.–Interviewee #1
		Our disease profile is in such a state that we cannot afford to wait any longer to really advocate at scale for breastfeeding.–Interviewee #12
		Advocacy needs to continue from various sectors to intensify this campaign and also to bring awareness to our healthcare providers and our committee with regards to what is happening and how all these things affect their ability to improve feeding of their children. Interviewee #13

#### Theme 1 –WHO guidelines on HIV and infant feeding

Informants stated that the many iterations of the WHO infant feeding guidelines for women living with HIV beginning 2001 undermined confidence in breastfeeding in South Africa as HIV positive women were initially told to avoid breastfeeding, then breastfeed for short periods and switch to formula and finally breastfeed if on antiretroviral therapy [1, 11–13]. According to participants, these shifts in policies and widespread distribution of formula at health facilities created uncertainty and confusion among health care workers and mothers in South Africa.

*Sub-theme 1a –Poor Code enforcement*. In 2011, the Government of South Africa out of concern for rising infant and child mortality and low exclusive breastfeeding (EBF) rates outlined its commitment to breastfeeding through the Tshwane Declaration to demonstrate its support for protecting and promoting EBF. The declaration included provisions to enact regulations to enforce the WHO Code, stated that mothers with HIV should breastfeed for 12 months, all public health facilities be baby-friendly by 2015, and no free distribution of formula (except by prescription) at government health facilities beginning 2012. Regulation 991 (R991) enacted under the 1972 “Foodstuffs, Cosmetics and Disinfectants Act” was legislated in December 2012 as the “Regulations relating to foodstuffs for infants and young children” to enforce the Code against marketing, promotion and sales of breastmilk substitutes (BMS) and designated commodities that displace breastfeeding for infants aged 0 to 36 months [26].

Despite these regulations and policies, the experts indicated that enforcement of the Code is “poorly implemented” and “weak” as there is no system, and there’s inadequate human and financial resources for monitoring and evaluation. Currently, just one person (Code compliance Officer) in the National Department of Health (DOH) is responsible for R991. The mechanism for reporting also needs to be updated and disseminated as it requires a form to be completed and uploaded to the DOH webpage. However, the DOH representatives indicated that they are upgrading their systems and will be collaborating with UNICEF to develop an App for reporting violations.

Another area of concern is that the BMS Industry is taking advantage of loopholes in the Code by shifting their promotions to social media (Facebook, What’s App, Blogs). They are also exploiting the marketing of formula, using cross-promotion tactics for children ages 3 years and up on these platforms and other media. Experts called for updating the Code to encompass the new digital media.

While few violations are occurring at public health facilities and institutions, they continue to occur frequently at private health facilities (pediatricians, gynecologists). According to the experts the ability to reign-in industry has been hampered because they have a strong foothold in South Africa and the financial resources to facilitate these activities. They also have resorted to legal threats when confronted or exposed. These actions and a lack of serious fines and prosecutions for violations have emboldened industry to forge ahead and breach the Code.

*Sub-theme 1b –Political will to enforce the Code*. While a majority of the experts felt that there was strong political will from current and past Ministers of Health and the current President’s wife to support breastfeeding and the Code, others felt that it was either not or a low priority since the resources (system, financial, human) needed were not in place for implementation and scale-up.

#### Theme 2 –Improving exclusive breastfeeding

The experts opined that the key areas of focus needed to improve EBF include maternity protections and community support, behavior-change communication (BCC) campaigns, training, and research.

*Sub-theme 2a - Maternity protection and support*. Although employees in the public sector get four months paid maternity leave, WHO guidelines recommend EBF for the first 6 months, as breastfeeding is likely to end when women return to the workplace. The government also needs to establish policies to cater for women employed in informal sectors (domestics, agricultural workers) since they do not get paid maternity leave.

Community support post-delivery is vital as women spend only a few hours in the hospital after birth they may not have a successful establishment of lactation unless they receive further support once discharged. The experts highlighted that women need support in the home, workplace, and other settings as well as an environment conducive to breastfeeding. Support should also come in the form of targeting household family members with influence on the mother such as partners and grandmothers. This type of support can be provided by community health workers and/or peers that are well trained and receive strong and consistent supportive supervision.

Another issue of concern is that many women live in food insecure households. South Africa has high levels of unemployment and single mothers, so many women need food assistance. Informants reported that food insecurity can affect breastfeeding because women may feel that their breastmilk is of poor quality, hence becoming more likely to use formula.

*Sub-theme 2b - Behavior-change communication campaigns*. While South Africa has had behavior change communications campaigns such as Side-by-Side, Road to Health booklet, Mom Connect, the experts felt that information for women on early infant and young child feeding should be complemented with a year-round communications campaign designed with guidance from behavior change communications specialists on the importance of EBF and breastfeeding beyond six months using a wide variety of communication channels and platforms.

Cultural practices were also reported to negatively impact breastfeeding, such as introducing liquids (water, teas) and solid foods early in the infant’s life. Other barriers include beliefs that women should stop breastfeeding to have sexual relations. In addition, perceptions that only poor people breastfeed and infant formula is best to help the baby grow are other factors negatively affecting breastfeeding uptake.

*Sub-theme 2c - Training and research*. The experts were all in agreement that in- and pre-service and ongoing education and trainings of all cadres of health care workers were essential to ensure consistent factual messaging and the necessary skills to support mothers with their breastfeeding goals. The experts highlighted that health care staff particularly nurses and doctors were not receiving updated training and support on WHOs infant feeding guidelines and recommend that all cadres of health workers receive training including on ethics. Also, of importance is to standardize the messaging on breastfeeding that is conveyed to mothers to be “less emotive” and more “evidence-based”. In terms of research, studies are needed to understand why some women are still not breastfeeding. Moreover, the data generated should be translated into policy and practice. It was noted that research has demonstrated low-cost interventions to increase breastfeeding practices, and they should be implemented in the country.

#### Theme 3 –Advocacy

Civil society groups in South Africa have been very active in promoting breastfeeding and the Code using a variety of methods and tactics. Civil society has actively identified, reported and addressed violations of R991 using public exposure of violations as a method to enforce R991 compliance [27].

*Sub-theme 3a - Actions by civil society and other stakeholders*. Breastfeeding activists in South Africa have been successful in halting industry violations of the Code and the need for conducive environments for women to breastfeed by conducting activities such as “flash boob mobs” protests at businesses that do not allow breastfeeding on their premises. Experts emphasized the need for strong advocacy to further raise awareness on Code violations and support for breastfeeding. They mentioned that it is important to not only document and report these violations but for academics to publish these violations in academic journals and highlight their findings at conferences and other forums. Another area of concern was the competition among advocacy groups by working in silos. It was recommended that all advocates work together particularly with the Government to raise awareness about these issues.

### The Breastfeeding Gear Model

The breastfeeding gear model (BFGM) is a framework for empowering policy makers to effectively scale up and sustain national breastfeeding programs [23]. This evidence-informed model [23, 28] consists of eight gears (advocacy; political will; legislation and policy; funding and resources; training and program delivery; promotion; research and evaluation; and coordination goals and monitoring). We mapped our thematic findings on this framework to highlight key multi-component strategies that were identified in the study to enforce the Code in the context of breastfeeding promotion, protection, and support.

#### Breastfeeding Gear Model as a tool to advance Code implementation and enforcement

The key recommendations by the experts to support the Code using the Gear Model were as follows (Table 3).

**Table 3:** Key recommendations to support the Code using the Gear Model.

**Advocacy gear**
• Maternity protection and support for working women, particularly for workers in informal sectors
• Strong monitoring and evaluation systems
• Raise awareness and document Code violations
• Support breastfeeding friendly (enabling) environments, e.g., workplaces, businesses, restaurants
• Community-based support systems for breastfeeding women
**Political will gear**
• Strong support for breastfeeding, but commitment needed to implement and enforce the Code at all levels of Government
• Elevate Code as a priority issue at all levels of Government with associated resources for scale-up
**Legislation and Policies gear**
• Policies to address conflict of interest at academic, health and other institutions
• Establish stronger penalties for Code violators
• Amend Code to include social and other digital platforms
• Update the infant and young child feeding (IYCF) policy with elements of the Code
• Update R991 legislation for Code since established 10 years ago
**Funding and Resources gear**
• Funding and resources needed to successfully implement and monitor the Code
• Funding for behavior change communications campaigns for breastfeeding protection, promotion, and support
**Training and Program delivery**
• Pre- and In-service training for all cadres of healthcare staff
• Include R991 regulations and Code in training curricula
• Training on conflict-of-interest issues related to the Code
• Refresher training for all healthcare workers
• Budget allocations for staff training
• Address cultural beliefs and practices that pose a barrier to breastfeeding
**Promotion gear**
• Standardized messaging to improve exclusive breastfeeding rates
• Multisectoral and multi-pronged approach e.g., involvement of churches, traditional healers in breastfeeding promotion and support campaigns
**Research and Evaluation gear**
• Translate research evidence into policy and practice
• Identify barriers on reasons for not breastfeeding/short breastfeeding time
• Research on impact of the Code on breastfeeding practices
• Strong monitoring and evaluation systems for Code
**Coordination, Goals and Monitoring gear**
• Strengthen implementation, monitoring and evaluation of the Code
• Establish year-round breastfeeding promotion campaigns
• Monitoring framework for digital platforms

## Discussion

The objective of this study was to gain an understanding of priority actions and strategies needed to improve breastfeeding outcomes in South Africa in the context of HIV. Some of the strengths of our study include the use of expert sources with both many years of work experience in South Africa and knowledge of the WHO Code and infant and young child feeding guidelines. Our team of experts represented academia, government, the United Nations, and civil society. While there was consensus that the government had made important strides to improve breastfeeding rates through inter alia promulgation of the Code of Marketing of Breastmilk Substitutes via regulation R991, key informants stated that more needs to be done to protect, promote and support breastfeeding beginning with updating and strengthening Code regulations established almost 10 years ago [14, 29]. However, just updating R991 is not sufficient to improve exclusive breastfeeding rates in South Africa and beyond. The breastfeeding gear model [23] is a useful framework for countries to assess their breastfeeding programs that can also be used to help South Africa develop a roadmap for improving breastfeeding outcomes in the country highlighting the role of Code enforcement.

Our findings are similar to a study on knowledge, perceptions and practices among South Africa dieticians regarding R991 regulations [30], whereby dieticians stated that they failed to report violations due to time intensive process, inaction against violators, and not receiving feedback on status of reports. Multiple studies have reported industry circumventing the Code via social media and other digital media platforms [31–35]. Industry in Vietnam also marketed BMS products not covered by Code regulations, highlighting the need to update these regulations. While the authors highlighted Vietnam’s strong legal framework to protect and promote breastfeeding, they experienced similar issues found in South Africa on gaps in Code implementation, monitoring and enforcement.

In their most recent report on the Marketing of Breastmilk Substitutes, WHO, UNICEF and IBFAN highlighted that as of April 2020 just 70% (136/194) of member countries have established legislation to implement the Code [36]. For the 136 countries with legislation, 79 ban promotion of BMS in health facilities, 84 prohibit poster displays of products, 30 prohibit the use of gifts and other incentives to health staff, and only 19 have provisions preventing sponsorships of scientific and professional association meetings [36]. Some of the UN’s recommendations to address marketing of BMS include those identified by our key informants, such as updating the regulations to address gaps, adequate budget and human resource allocations for implementation, monitoring and enforcement framework for all relevant government agencies, penalties for violators that will serve as a deterrent, and training for healthcare staff particularly on conflicts of interest [36]. Finally, the UN agencies recommended expanding the types of products under the regulations to include follow-up formula, growing-up milks and complementary foods to encompass young children up to 36 months of age which have been endorsed by other authors [19, 37].

The recent guidance by UNICEF and WHO on improving breastfeeding highlights the importance of counselling as an important strategy to improve breastfeeding practices [38]. This issue was also flagged by our experts as integral to successful outcomes as they recommended the need for trained healthcare staff and community peer support after delivery. Food insecurity is another important area to be addressed as mothers who have poor or inadequate diets believe that they produce poor quality milk and refrain from breastfeeding as highlighted in other studies [39–41]. As grandparents, partners, and other elders can pose a challenge to initiating and maintaining EBF, behavior change communications campaigns should also include these individuals to facilitate better breastfeeding outcomes.

## Conclusion

The key informants identified issues that need to be addressed to improve breastfeeding outcomes in South Africa. Strong political will is a key ingredient to harness the resources (human, financial) needed to implement, monitor, and act against violators. South Africa and other countries with similar challenges should explore WHOs Network for Global Monitoring and Support for Implementation of the International Code of Marketing of Breast-milk Substitutes and Subsequent relevant World Health Assembly Resolutions (NetCode) [42, 43]. These two toolkits were developed for periodic and ongoing monitoring and assessment of the International Code of Marketing of Breastmilk Substitutes and have been pilot-tested in Cambodia [44] with promising results. These monitoring systems once established and maintained are useful tools to regulate the marketing of BMS and infant and young child foods and other products.

## Supporting information

S1 Questionnaire (DOCX)
